# Implementation of Standardized Care for the Medical Stabilization of Patients With Anorexia Nervosa

**DOI:** 10.1097/pq9.0000000000000582

**Published:** 2022-08-26

**Authors:** Taraneh Shafii, Alex Morrison, Pingping Qu, Lori Rutman, Ron Kaplan

**Affiliations:** From the * Division of Adolescent Medicine, Department of Pediatrics, University of Washington, Seattle Children’s Hospital, Seattle, WA; † Department of Pediatrics, University of Washington, Seattle Children’s Hospital, Seattle, WA; ‡ Biostatistics Epidemiology and Analytics for Research (BEAR) Core, Seattle Children’s Research Institute, Seattle, WA; § Division of Emergency Medicine, Department of Pediatrics, University of Washington; Seattle Children’s Hospital, Seattle, WA.

## Abstract

Approaches to refeeding patients with anorexia nervosa for medical stabilization vary across institutions, and there is no established standard of care. This study assessed the impact of a refeeding pathway on hospital length of stay and transfer to the psychiatry unit.

**Methods:**

This quality improvement intervention sought to standardize care for adolescents with anorexia nervosa at a tertiary care, free-standing children’s hospital from Spring 2017 to Fall 2018. The pathway specified admission criteria, nutritional advancement, activity restriction, laboratory monitoring, readiness to transfer to the psychiatry unit, and discharge criteria. Statistical process control analysis was utilized to identify system-level changes over time. We used linear regression to assess pre- and postpathway differences in length of stay and transfer to the psychiatry unit.

**Results:**

There were 161 patient encounters for anorexia nervosa admitted for medical stabilization. 84% of the sample were female with median age of 15.2 (IQR 14.0–17.0) years. There was no difference in hospital length of stay between the pre- and postpathway groups. There was a statistically significant increase in the proportion of patients transferred to the psychiatry unit over the study period.

**Conclusion:**

Clinical pathway use to deliver standardized care to achieve medical stability for patients with anorexia nervosa did not shorten hospital length of stay. Multiple potentially confounding medical and psychosocial factors may have contributed to this lack of improvement.

## INTRODUCTION

Adolescents with anorexia nervosa may restrict oral intake to the point of starvation and profound bradycardia, which can require hospitalization.^1–5^ Refeeding syndrome is a potentially life-threatening complication of acute nutritional rehabilitation. It is characterized by decreasing phosphorus levels, which, if not corrected can lead to diaphragmatic paralysis and cardiac arrhythmia.^6–8^ Patients may require medical and behavioral monitoring during refeeding. At present, there is no published national standardized recommendation for refeeding patients, and the approach varies by institution.^9^

There is no Adolescent or Eating Disorder Unit in our hospital, and these patients are not colocated, so there was variability among staff and providers in behavioral monitoring, feeding advancement, lab frequency, and discharge criteria. Medical floor limitations include lack of staff trained in eating disorder behaviors and lack of monitoring during and after meals. Patients could therefore engage in unobserved behaviors such as flushing food down the toilet, surreptitious exercise, or purging. These behaviors interfere with weight gain and increase the length of stay to reach medical stability.

In April 2017, we developed and implemented an eating disorder-refeeding pathway to standardize care for the medical stabilization of patients with eating disorders. Before pathway implementation, patients were not routinely transferred to the psychiatric unit and were only considered for transfer if their behaviors, such as food refusal and excessive exercise, were uncontrollable on the medical floor. Arranging transfer and negotiating the necessity with patients and families was challenging and required unsustainable hours of time from the interdisciplinary team.

The inpatient psychiatry unit provides care to youth with varying mental health problems, including aggression, depression, and suicidality. Patients with eating disorders may be admitted to the psychiatry unit on the General Medicine service if they are working toward medical stability. Treatment for eating disorders on the psychiatry unit includes staff trained in coaching through meals; behavioral supervision, including 1-hour bathroom lock-out after meals; and redirection for excessive body movement. Patients with eating disorders benefit from this combination of medical monitoring and trained behavioral support during refeeding.

## RATIONALE

**Fig. 1.**
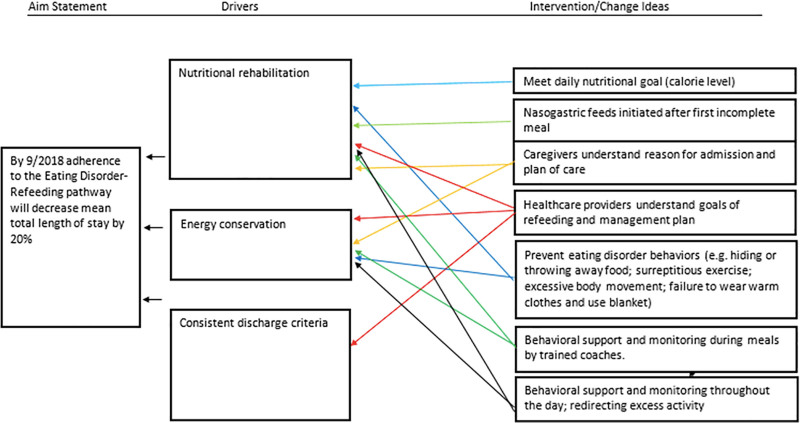
Key driver diagram.

The aim of the Eating Disorder-Refeeding Pathway was to safely streamline care and decrease hospital length of stay. The pathway included admission and discharge criteria, medical and behavioral monitoring, and optimal nutritional advancement. We anticipated the pathway would decrease the length of stay by reducing nutritional advancement variability, limiting allowed activity (eg, walking and wheelchair use), and standardizing admission and discharge criteria (Fig. 1).

## SPECIFIC AIMS

This study aimed to evaluate the impact of implementing standardized care via the Eating Disorder-Refeeding Pathway on hospital length of stay and transfer to the psychiatry unit. Specifically, by September 2018, we sought to decrease the mean total inpatient length of stay (LOS) for patients with eating disorders by 20%.

## METHODS

The setting was a tertiary, university-affiliated 350 bed, free-standing pediatric hospital. Patients are admitted to General Medicine teams with an attending physician, residents, interns, and medical students. Consultation is provided by adolescent medicine, child psychiatry, and nutrition. At the time of pathway implementation, we admitted approximately 90 patients per year with eating disorders for medical stabilization.

In the Spring of 2016, an interdisciplinary team of physicians, nurses, and dietitians collaborated with the hospital clinical effectiveness team to address the following question: What is the most effective approach to medically stabilize patients with eating disorders? We conducted a systematic literature review for information on admission and discharge criteria^4,10^; rate and method of refeeding^11–13^; risks of starvation and refeeding^14,15^; and any existing guidelines.^1–3,16^ We found no standard approach for medical stabilization and no definitive studies to answer these questions. We developed the Eating Disorder-Refeeding Pathway based on available studies, published guidelines, and expert opinion. Throughout pathway development, we obtained feedback from stakeholders involved directly in patient care. We used low fidelity simulation of a patient progressing through the pathway, from evaluation in the emergency department to readiness for discharge. Before implementation, we held sessions with residents and attendings to introduce the pathway. We created teaching modules for emergency medicine clinicians and hospitalists. As a pathway team, we met regularly to monitor metrics, adherence to the pathway, and needed modifications.

The pathway was implemented in April 2017 and was designed to incorporate both acute medical management and intensive behavioral support. This study included patients admitted from May 1, 2016, to September 30, 2018. To capture the patient population of interest, pre- and postpathway, we used criteria unique to managing these patients. Patients included had to meet all three of the following: (1) anorexia nervosa as primary or secondary diagnosis; (2) admitted to General Medicine, and (3) three phosphorus levels obtained within the first 5 days of admission (which reflects monitoring for refeeding syndrome). We used these criteria to ensure patients in the study were admitted to the hospital for medical stabilization due to the sequelae of starvation from anorexia nervosa.

## INTERVENTION

The pathway had multiple phases of care. The Emergency Department phase outlined the initial evaluation and need for hospitalization (see Appendix, Supplemental Digital Content 1, available at http://links.lww.com/PQ9/A390). Hospital admission initiated the Medical Unit phase (see Appendix, Supplemental Digital Content 2, available at http://links.lww.com/PQ9/A391) for standardized vital sign measurement, nutritional progression (including criteria for nasogastric feeds), and frequency of electrolyte monitoring. Adolescent medicine, psychiatry and nutrition services were consulted, and family care conferences were initiated, as were biweekly provider care conferences.

To benefit from the expertise of staff trained in eating disorders, the pathway recommended the transfer of patients to a medical bed on the psychiatry unit by hospital day 5. Once patients demonstrated all heart rates >30 beats per minute (HR monitor and manual pulse check) for 48 hours, had stable electrolytes, and no other acute medical problems requiring immediate intervention such as hemodynamic instability or refeeding syndrome, they could transfer to the psychiatric unit to continue medical stabilization. Transfer initiated the Medical Behavioral Bed (a bed on the psychiatry unit equipped for medical monitoring) phase (Appendix, Supplemental Digital Content 3, available at http://links.lww.com/PQ9/A392,4 http://links.lww.com/PQ9/A393). The nutritional progression and laboratory monitoring were the same as the Medical Unit phase with the additional orders of 5-minute only showers, bathroom lock-out 1 hour after meals, and activity restrictions. The recommended discharge criteria for medical stabilization and follow-up care were the same regardless of whether on the Medical or Psychiatric Unit.

## STUDY OF THE INTERVENTION

This quality improvement study utilized statistical process control to identify changes over time. For all centerline shifts, we followed the standard rules for identifying special cause variation. All control charts were created using the QI Charts 2.0 add-on for Microsoft Excel (Process Improvement Products, Austin, TX).

We performed a retrospective chart review with a study period of 12 months preintervention and 18-months postintervention for comparison. From the time of implementation, there was an average pathway utilization rate of 97%. For additional verification, these patients were on the pathway, we limited postpathway data to include those patients who had pathway-specific laboratory orders of daily phosphorus levels during the first 5 days of admission. We used linear regression to assess differences in pre- and postpathway populations (Table 1).

**Table 1.** Encounter-level Demographics.

Variable	n	Level	Overall	Prepathway	Postpathway	*P*
^*^Age	161	median [IQR]	15.2 [14.0–17.0]	14.5 [13.7–17.0]	15.6 [14.2–17.0]	0.012
Gender	161	Female	136 (84%)	57 (85%)	79 (84%)	1.0
		Male	25 (16%)	10 (15%)	15 (16%)	
Race	147	White or Caucasian	99 (67%)	47 (76%)	52 (61%)	0.091
		Other	48 (33%)	15 (24%)	33 (39%)	
Ethnicity	150	Non-Hispanic	113 (75%)	53 (82%)	60 (71%)	0.177
		Hispanic	37 (25%)	12 (18%)	25 (29%)	
Language	161	English	131 (81%)	55 (82%)	76 (81%)	1.0
		Other	30 (19%)	12 (18%)	18 (19%)	
Insurance	161	Commercial	86 (53%)	38 (57%)	48 (51%)	0.583
type		Medicaid/Govt	75 (47%)	29 (43%)	46 (49%)	

^*^P < 0.05.

## MEASURES

The primary outcome measure was total hospital length of stay (medical floor and psychiatry unit). Prepathway patients were not expected to transfer to the psychiatry unit. Postpathway patients were expected to transfer to the psychiatry unit by day 5. The primary discharge criteria was medical stability with HR > 45 for 24 hours regardless of bed location (see Appendix, Supplemental Digital Content 5, available at http://links.lww.com/PQ9/A394). Length of stay was calculated in days. The proportion of patients transferred to a medical behavioral bed on the psychiatry unit was a process measure (Fig. 4). With specialty behavioral monitoring, we expected patients to reach medical stability efficiently and thus decrease overall hospital length of stay.

**Fig. 2.**
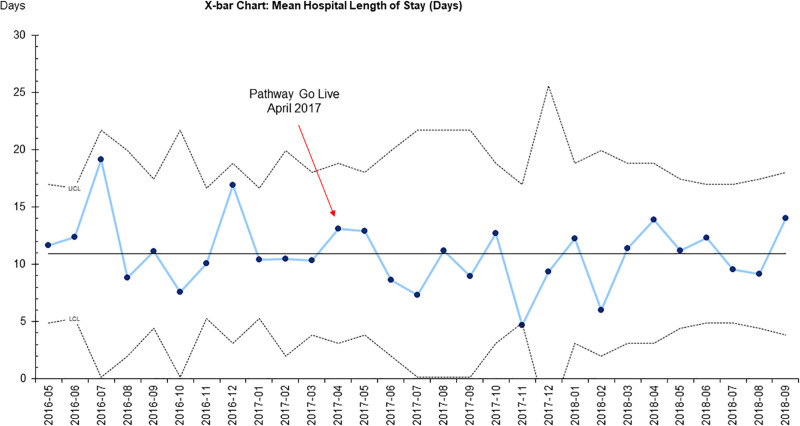
X-bar chart for mean hospital length of stay. *x* axis: month and year; *y* axis: days.

**Fig. 3.**
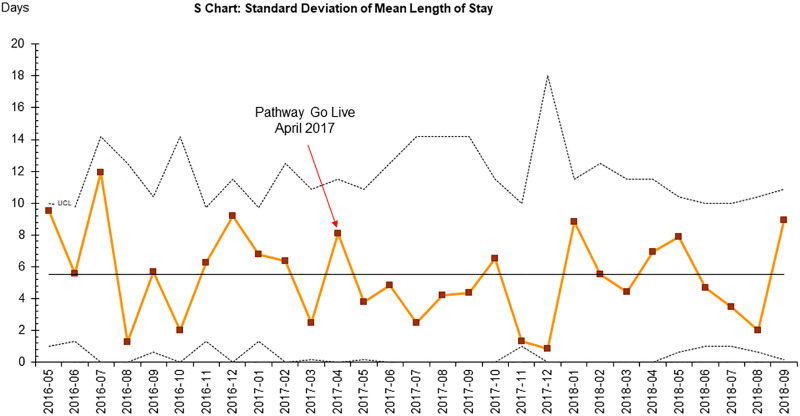
S-chart for SD of mean length of stay. *x* axis: month and year; *y* axis: days.

**Fig. 4.**
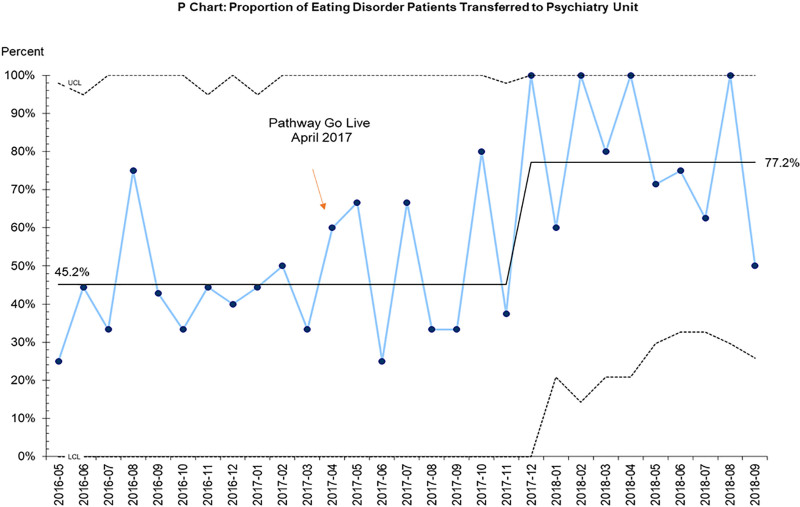
P-chart for proportion of patients with eating disorders transferred to psychiatry unit. *x* axis: month and year; *y* axis: proportion of patients.

As length of stay may have been influenced by the inability to transfer to the psychiatry unit, we compared patients meeting transfer criteria who were transferred to the unit by hospital day 5 to those patients meeting transfer criteria and remained on the medical floor. To conservatively identify pre- and postpathway patients meeting transfer eligibility, we identified objective variables from the medical record. Transfer eligibility criteria were defined as HR >30 for 48 hours; BMI z score –2.0 or greater; stable electrolytes; and younger than 18 years old. Lowest heart rate was defined as sleeping heart rate nadir in the first 48 hours of admission.

## ETHICAL CONSIDERATIONS

This study was approved via expedited review by Seattle Children’s Hospital institutional review board.

## ANALYSIS

Demographic differences between pre- and postintervention groups were assessed using descriptive statics with R version 4.0.3. Linear regression was used to compare pre- and postpathway populations for (1) total hospital LOS and (2) proportion transferred from medical floor to a medical behavioral bed (MBB) in the psychiatry unit, with unadjusted models and adjusted models controlling for transfer eligibility criteria as defined above.

For the postpathway population only, linear regression was used to assess LOS of patients transferred to an MBB when they met criteria by day 5 compared with LOS of patients not transferred to an MBB when meeting criteria with models unadjusted and adjusted for transfer eligibility criteria.

We used statistical process control (SPC) methodology to evaluate the impact of pathway implementation on length of stay and transfer to the psychiatric unit. All control charts in this study were created using the QI Charts 2.0 add-on for Microsoft Excel (Process Improvement Products, Austin, TX).

### Demographics Characteristics

There were 161 patient encounters meeting eligibility criteria. Patients pre- and postintervention were similar in demographic characteristics except for the postpathway group being older (15.6 years; IQR 14.2–17.0) than prepathway (14.5 years; IQR 13.7–17.0) (*P* = 0.012). Most patients were female (136/161, 84%) and White (99/147, 67%). Patients in the study were similarly divided between commercial (86/161, 53%) and government insurance 75/161 (47%) (Table 1).

### Medical Acuity

Medical acuity between pre- and postpathway patients was similar and both groups demonstrated severe malnutrition and severe bradycardia. In both groups, the proportion of patients presenting with electrolyte abnormalities was similar. The proportion of patients requiring phosphorus supplementation during refeeding also did not differ between groups at 20% (32/161) (Table 2).

**Table 2.** Medical Acuity and Outcomes.

Variable	Level	Overall	Prepathway	Postpathway	*P*
		N = 161	N = 67	N = 94	
Lowest heart rate first 48 hours	Mean (SD)	39.9 (9.2)	38.9 (6.1)	40.7 (10.8)	0.216
BMI (z score)	Mean (SD)	–1.8 (1.4)	–1.6 (1.3)	–1.9 (1.5)	0.176
Low WBC < 5.0 (N = 133)	Normal	62 (47%)	27 (55%)	35 (42%)	0.187
	Low	71 (53%)	22 (45%)	49 (58%)	
Low Hgb < 11.9 (N = 133)	Normal	116 (87%)	40 (82%)	76 (90%)	0.228
	Low	17 (13%)	9 (18%)	8 (10%)	
Phosphorous supplement administered	No	129 (80%)	53 (79%)	76 (81%)	0.941
	Yes	32 (20%)	14 (21%)	18 (19%)	
Hospital length of stay (days)	mean (SD)	11 (6.2)	11.5 (6.8)	10.6 (5.8)	0.349
Met transfer Criteria^†^ by hospital day 5	No	96 (60%)	35 (52%)	61 (65%)	0.147
	Yes	65 (40%)	32 (48%)	33 (35%)	
^*^Transferred to psychiatric unit	No	70 (43%)	39 (58%)	31 (33%)	0.005
	Yes	91 (57%)	28 (42%)	63 (67%)	

*P < 0.05.

†Transfer criteria by hospital day 5 defined as HR > 30 for 48 hours; BMI z-score –2.0 or greater; stable electrolytes (not on supplement); less than 18 years old (pre-existing requirement of psychiatry unit); or if transferred to psychiatry unit before hospital day 5.

## OUTCOMES

### Length of Stay

The mean LOS for this project was 10.6 days, as depicted in Figures 2 and 3. There was no difference in length of stay pre- or postpathway in unadjusted or adjusted multivariate linear regression models controlling for age, BMI, lowest heart rate, and need for phosphorus supplementation.

### Transfer to Psychiatry Unit

A statistically significant increased number of patients were transferred to the psychiatry unit postpathway (63/94 67%) as compared to prepathway (28/67 42%) (*P* = 0.003). The timing of this difference was further codified by the upward centerline shift (SPC p-chart; Fig. 4); however, this increase began seven months after pathway implementation.

### Length of Stay and Transfer Criteria

In unadjusted and adjusted multivariate linear regression models, there was no difference in length of stay pre- or postpathway for patients transferred to the psychiatry unit by day 5.

## DISCUSSION

As implemented at our institution, a standardized care pathway for hospitalized patients with eating disorders did not decrease hospital length of stay. This pathway did not achieve its intended aim. There was an increase in the number of patients transferred to the psychiatry unit from the general medical units. This change did not occur until 7 months after pathway implementation and therefore it is unclear if the pathway had its intended effect on the transfer rate.

We hypothesized that for hospitalized patients with eating disorders, a standardized care pathway with criteria for nutritional advancement, laboratory test timing, and discharge criteria would decrease hospital length of stay because care would be less variable between providers. In effect, this was an attempt to simulate elements of an eating disorder unit on the medical floor without the structure and staffing of a specialized unit. However, care for eating disorders is complex and standardization (pathway) does not fully consider patient compliance with care, family understanding of the illness and needed treatment, and availability of ongoing care after discharge.

We hypothesized early transfer to the psychiatry unit would facilitate more efficient treatment and shorter lengths of stay. The pathway care model recommended all patients be transferred to the psychiatry unit by hospital day 5. As can occur with process change in QI projects, there were multiple unanticipated patient transfer barriers for months after implementation, which outline the limitations of this study. First, there was significant family resistance and refusal to transfer their child to the psychiatry unit for care, especially among families admitted previously when the psychiatry unit was not part of the pathway. Pediatric providers felt unprepared to advocate for the need for transfer to families, and in September 2017, the psychiatry team provided a job aid for how to facilitate difficult conversations. We believe this barrier could have been anticipated, but the job aid helped mitigate this family challenge. Unfortunately, we do not have data on the proportion of caregivers declining transfer to the psychiatric unit and this could be a source of confounding.

Second, within a few months of pathway implementation, there was an increased community need for hospitalization of patients with high mental health acuity (eg, aggression and suicidality) which, combined with a staffing shortage on the psychiatry unit, the 6 psychiatric beds equipped for medical patients were often filled with other mental health patients. As patients with eating disorders do not require locked units, they remained on the medical floor. The structure of our hospital units and staffing model, with a separation of medical from behavioral monitoring does not provide the combined care needed for patients with eating disorders. As we were developing the pathway, in anticipation of barriers in admitting to the psychiatric unit, we met with hospital leadership to explore and advocate for 4 to 6 beds colocated in an area of the hospital that could provide both medical and behavioral monitoring; however, the hospital was unable to resource the request.

To understand why overall length of stay was not decreased, even for patients transferred to the psychiatry unit, we need to consider several factors. Patients transferred to the psychiatric unit may have had more severe eating disorders than those remaining on the medical floor. An eating disorder may be considered more severe if there are more significant medical sequalae such as lower percentage of treatment goal weight and lower heart rate or if more entrenched in eating disorder behaviors thus more successful at surreptitious exercising or food riddance. Patients are not on bed rest on the psychiatry unit and the associated increase in energy expenditure may have canceled out the benefit of behavioral monitoring. In addition, length of stay data may have been skewed by more severe patients discharging days after meeting discharge criteria of HR > 45 for 24 hours if they were awaiting placement in residential or partial hospitalization programs.

Our institution has strong QI support and a robust program for developing standardized clinical work. Pathways are designed by interdisciplinary teams of clinicians who provide direct patient care, and there is a robust implementation process, including in-person education and electronic teaching modules for providers. Despite this, our pathway failed to achieve the desired aims. This may have been due to problems with the pathway content, implementation strategy, or the multifaceted complexities of eating disorders.

Since development of our pathway, there has been one publication of a comprehensive refeeding pathway for medical stabilization, which is very similar to ours concerning patients’ location on the medical floor, the approach to nutritional rehabilitation, medical monitoring, behavioral observation, psychotherapeutic support, and interdisciplinary care.^18^ Their goal was to accelerate refeeding without increasing safety risks, and their reported length of stay of 11 days did not change, which is similar to our findings. Other studies have been published about initiating refeeding at higher calorie levels to assess safety, risk of refeeding syndrome,^19–26^ and impact on the length of stay.^7,27–29^ Accelerated refeeding is defined differently among various studies; however, due to the wide variation in medical acuity of our patients, we initiated 1200 kcal on admission and within 24 hours adjusted to 1500 to 1800 kcal increasing by 200 kcal/d to goal once the dietitian obtained the nutritional history.

Pathway utilization continues and we believe brings value for several reasons: (1) it is a vehicle to provide equitable care across a spectrum of patients; (2) through quarterly meetings and regular PDSA cycles, the pathway is living document that is modified as circumstances change; and (3) families often express appreciation for the robust interdisciplinary team providing care to their child. In sum, while our pathway as implemented did not achieve its intended aim, it remains a valuable mechanism for reducing unnecessary care variation among a disparate and medically complex group of patients. Further, there are many pathway elements that may impact length of stay and warrant exploration. Future evaluations may include modifications regarding starting calorie intake and incremental daily calories adjustments to reach goal more quickly.

## FUNDING

Funding for data acquisition and statistical analysis was provided through the Seattle Children’s and University of Washington Quality Improvement Scholars Program.

## DISCLOSURE

The authors have no financial interest to declare in relation to the content of this article.

## Supplementary Material



## References

[1] Junior MARSIPAN Group. *Junior MARSIPAN: management of really sick patients under 18 with anorexia nervosa, college report CR 168*. Royal College of Psychiatrists; 2012. https://www.sisdca.it/public/pdf/Junior-MARSIPAN--Management--of-Really-Sick-Patients-under-18--with-Anorexia-Nervosa.pdf. Accessed June 14, 2016.

[2] Royal College of Psychiatrists. CR189: *MARSIPAN: Management of really sick patients with anorexia nervosa*. Royal College of Psychiatrists; 2014. https://www.rcpsych.ac.uk/docs/default-source/improving-care/better-mh-policy/college-reports/college-report-cr189.pdf?sfvrsn=6c2e7ada_2. Accessed June 14, 2016.

[3] National Institute for Health and Care Excellence (NICE). Eating disorders recognition and treatment, NICE guideline. NICE; 2017. www.nice.org.uk/guidance/ng69. Accessed July 19, 2016. 28654225

[4] Golden NH Katzman DK Sawyer SM. Update on the medical management of eating disorders in adolescents. J Adolesc Health. 2015;56:370–375. 25659201 10.1016/j.jadohealth.2014.11.020

[5] Hofer M Pozzi A Joray M. Safe refeeding management of anorexia nervosa inpatients: an evidence-based protocol. Nutrition. 2014;30:524–530. 24698345 10.1016/j.nut.2013.09.019

[6] Brown CA Sabel AL Gaudiani JL. Predictors of hypophosphatemia during refeeding of patients with severe anorexia nervosa. Int J Eat Disord. 2015;48:898–904. 25846384 10.1002/eat.22406

[7] Golden NH Keane-Miller C Sainani KL. Higher caloric intake in hospitalized adolescents with anorexia nervosa is associated with reduced length of stay and no increased rate of refeeding syndrome. J Adolesc Health. 2013;53:573–578. 23830088 10.1016/j.jadohealth.2013.05.014

[8] Mehanna HM Moledina J Travis J. Refeeding syndrome: what it is, and how to prevent and treat it. BMJ. 2008;336:1495–1498. 18583681 10.1136/bmj.a301 PMC2440847

[9] Norris ML. Editorial: Phosphate supplementation during refeeding of hospitalized adolescents with anorexia nervosa – watch and wait or empirically treat. J of Adol Health. 2016; 58:593–594. 10.1016/j.jadohealth.2016.03.030 27210006

[10] Ebeling H Tapanainen P Joutsenoja A; Finnish Medical Society Duodecim. A practice guideline for treatment of eating disorders in children and adolescents. Ann Med. 2003;35:488–501. 14649331 10.1080/07853890310000727

[11] Garber AK Sawyer SM Golden NH. A systematic review of approaches to refeeding in patients with anorexia nervosa. Int J Eat Disord. 2016;49:293–310. 26661289 10.1002/eat.22482 PMC6193754

[12] Herpertz-Dahlmann B van Elburg A Castro-Fornieles J. ESCAP Expert Paper: New developments in the diagnosis and treatment of adolescent anorexia nervosa–a European perspective. Eur Child Adolesc Psychiatry. 2015;24:1153–1167. 26226918 10.1007/s00787-015-0748-7 PMC4592492

[13] Suárez-Pinilla P Peña-Pérez C Arbaizar-Barrenechea B. Inpatient treatment for anorexia nervosa: a systematic review of randomized controlled trials. J Psychiatr Pract. 2015;21:49–59. 25603451 10.1097/01.pra.0000460621.95181.e2

[14] Sachs KV Harnke B Mehler PS. Cardiovascular complications of anorexia nervosa: A systematic review. Int J Eat Disord. 2016;49:238–248. 26710932 10.1002/eat.22481

[15] O’Connor G Nicholls D. Refeeding hypophosphatemia in adolescents with anorexia nervosa: a systematic review. Nutr Clin Pract. 2013;28:358–364. 23459608 10.1177/0884533613476892 PMC4108292

[16] Hay P Chinn D Forbes D; Royal Australian and New Zealand College of Psychiatrists. Royal Australian and New Zealand College of Psychiatrists clinical practice guidelines for the treatment of eating disorders. Aust N Z J Psychiatry. 2014;48:977–1008. 25351912 10.1177/0004867414555814

[17] Provost LP Murray SK.The Health Care Data Guide: Learning from Data for Improvement. San Francisco, CA: Jossey-Bass. 2011.

[18] Peebles R Lesser A Park CC. Outcomes of an inpatient medical nutritional rehabilitation protocol in children and adolescents with eating disorders. J Eat Disord. 2017;5:7. 28265411 10.1186/s40337-017-0134-6 PMC5331684

[19] Davis C Hong WJN Zhang SL. Outcomes of a higher calorie inpatient refeeding protocol in Asian adolescents with anorexia nervosa. Int J Eat Disord. 2021;54:95–101. 33159492 10.1002/eat.23403

[20] Hass V Kohn M Korner T. Practice-based evidence and clinical guidance to support accelerated re-nutrition of patients with anorexia nervosa. J Amer Acad Child Adole Psych. 2020;60:555–561. 10.1016/j.jaac.2020.09.010 PMC10863999 32998025

[21] O’Connor G Nicholls D Hudson L. Refeeding Low Weight Hospitalized Adolescents With Anorexia Nervosa: A Multicenter Randomized Controlled Trial. Nutr Clin Pract. 2016;31:681–689. 26869609 10.1177/0884533615627267

[22] Smith K Lesser J Brandenburg B. Outcomes of an inpatient refeeding protocol in youth with Anorexia Nervosa and atypical Anorexia Nervosa at Children’s Hospitals and Clinics of Minnesota. J Eat Disord. 2016;4:35. 28018595 10.1186/s40337-016-0124-0 PMC5165845

[23] Maginot TR Kumar MM Shiels H Kaye M Rhee K. Outcomes of an inpatient refeeding protocol in youth with anorexia nervosa: rady children’s hospital san diego/ university of california, san diego. J of Eat Disord. 2017; 5:1–10. 28053702 10.1186/s40337-016-0132-0 PMC5209953

[24] Leitner M Burstein B Agostino H. Prophylactic phosphate supplementations for the inpatient treatment of restrictive eating disorders. J Adol Health. 2016; 58:616–620. 10.1016/j.jadohealth.2015.12.001 26774639

[25] Madden S Miskovic-Wheatley J Clarke S Touyz S Hay P Kohn M. Outcomes of rapid refeeding protocol in adolescent anorexia nervosa. J of Eating Disorders. 2015; 3:1–9. 10.1186/s40337-015-0047-1 PMC4379764 25830024

[26] Redgrave GW Coughlin JW Schreyer CC. Refeeding and weight restoration outcomes in anorexia nervosa: Challenging current guidelines. Int J Eat Disord. 2015;48:866–873. 25625572 10.1002/eat.22390

[27] Garber AK Mauldin K Michihata N. Higher calorie diets increase rate of weight gain and shorten hospital stay in hospitalized adolescents with anorexia nervosa. J Adolesc Health. 2013;53:579–584. 24054812 10.1016/j.jadohealth.2013.07.014 PMC4452504

[28] Garber AK Cheng J Accurso EC. Short-term outcomes of the study of refeeding to optimize inpatient gains for patients with anorexia nervosa: a Multicenter Randomized Clinical Trial. JAMA Pediatr. 2021;175:19–27. 33074282 10.1001/jamapediatrics.2020.3359 PMC7573797

[29] Lascar R Letranchant A Hirot F. [What factors explain the length of hospitalization for anorexia nervosa: A systematic review]. Encephale. 2021;47:362–368. 33752870 10.1016/j.encep.2020.11.002

