# Supplementary figures and images for: Implementation of Standardized Care for the Medical Stabilization of Patients With Anorexia Nervosa
Source: Pediatr Qual Saf. 2022 Aug 26;7(5):e582. doi: 10.1097/pq9.0000000000000582 (PMC9416762; doi:10.1097/pq9.0000000000000582)

## Appendix 1. Emergency Department Phase of Standard Work Pathway

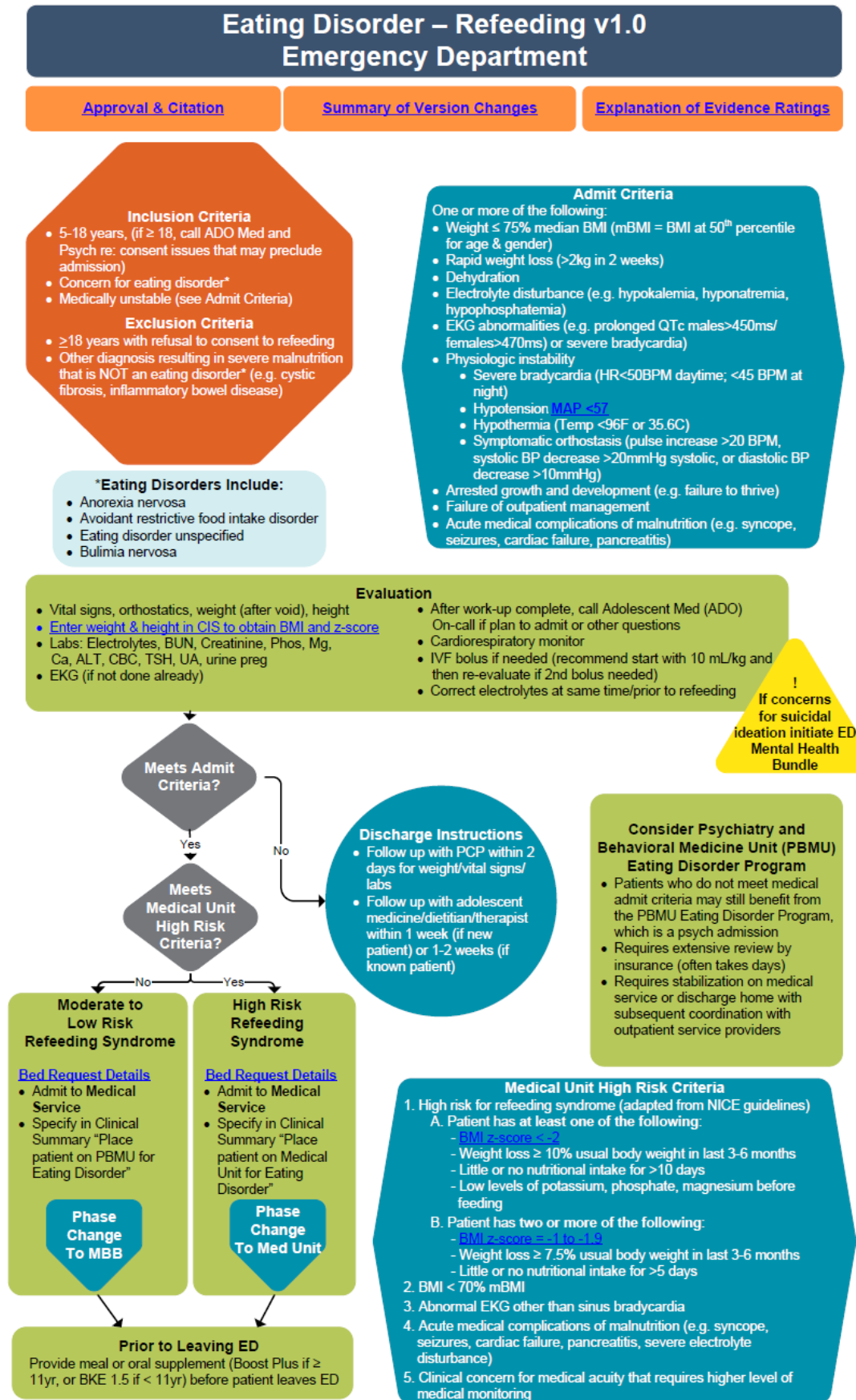

Supplement: Supplementary file 1 [file pqs-7-e582-s001.pdf]

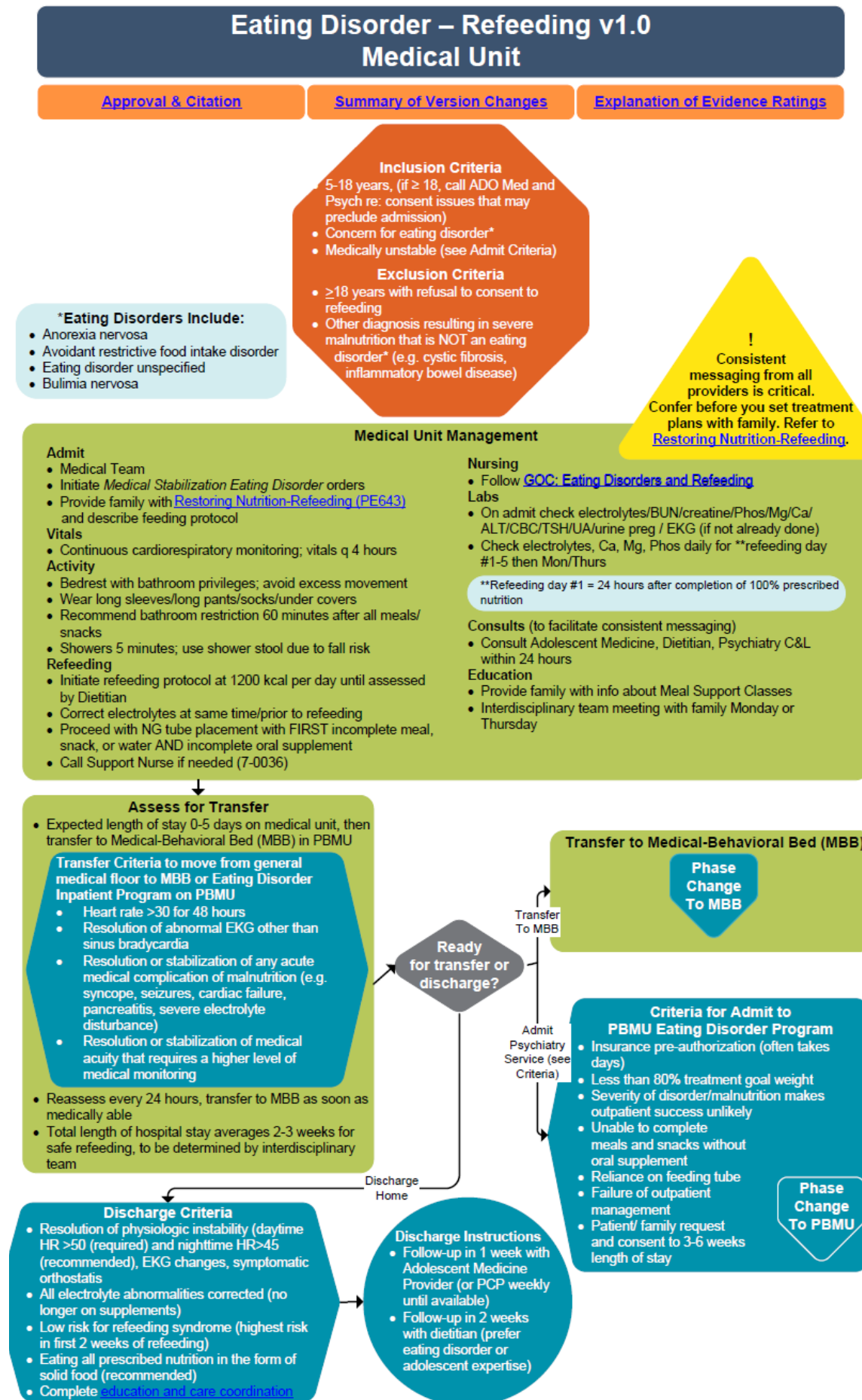

Supplement: Supplementary file 2 [file pqs-7-e582-s002.pdf]

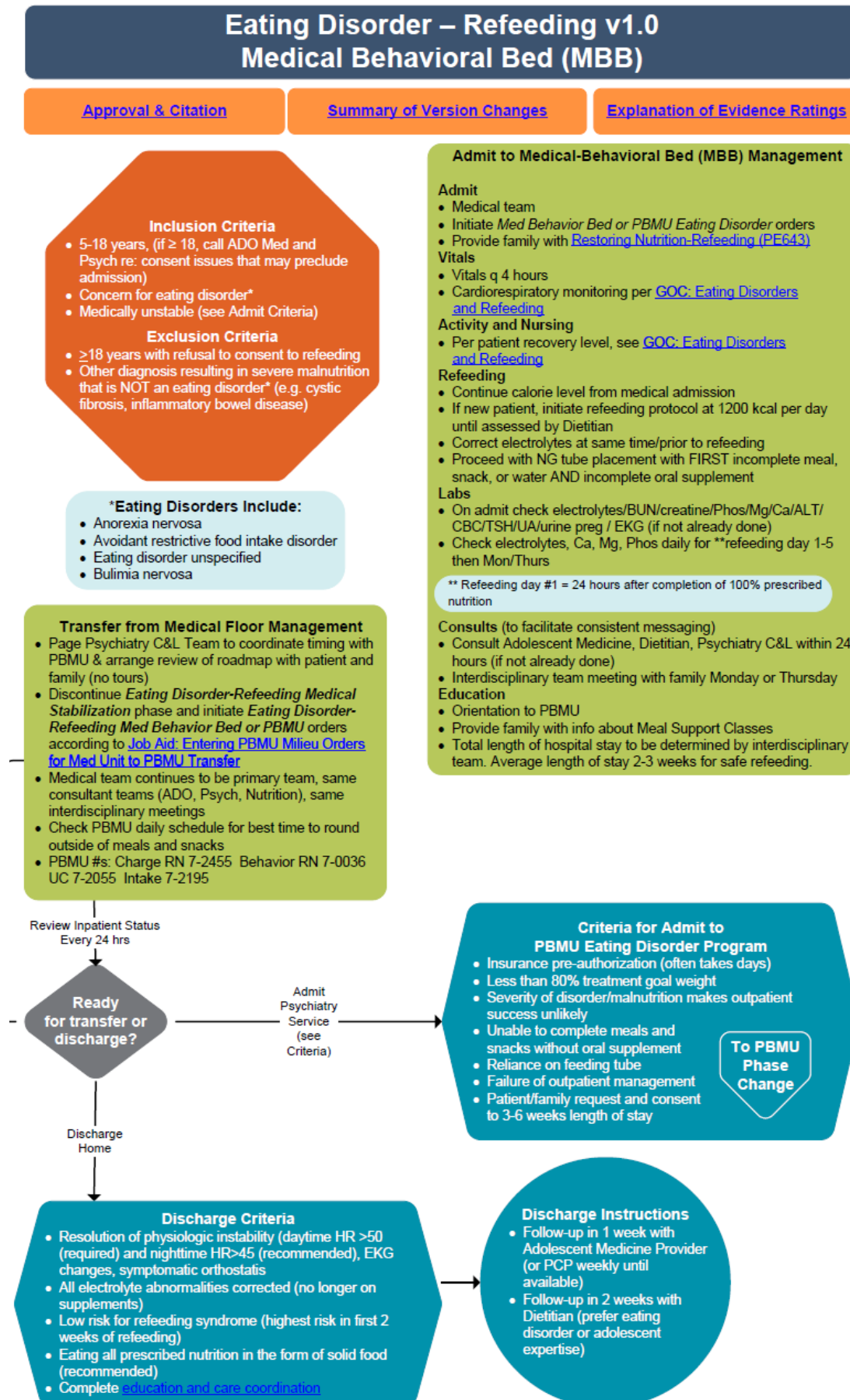

Supplement: Supplementary file 3 [file pqs-7-e582-s003.pdf]
