# Supplementary material for: Implementation of Standardized Care for the Medical Stabilization of Patients With Anorexia Nervosa
Source: Pediatr Qual Saf. 2022 Aug 26;7(5):e582. doi: 10.1097/pq9.0000000000000582 (PMC9416762; doi:10.1097/pq9.0000000000000582)
Supplement: Supplementary file 4 [file pqs-7-e582-s004.pdf]

Appendix 4. Hospital Terminology and Definitions

| Hospital Term | Definition |
|---|---|
| CIS | Former version of hospital Electronic Medical Record |
| | |
| EDO Mental Health Bundle | Procedure activated when there is concern for patient with |
| | suicidality including 1:1 watch, mental health evaluation |
| | |
| PBMU | Psychiatry and Behavioral Medicine Unit |
| | This is the hospital psychiatric unit which cares for patients |
| | with a variety of mental health diagnoses |
| | |
| MBB on PBMU | Medical Behavioral Bed (MBB) a bed on the psychiatric unit |
| | for patients with medical *and* mental health needs. The |
| | room has the same equipment as those on the medical unit |
| | For the pathway if the patient is on a medical team for |
| | medical stabilization, they may be transferred to MBB for |
| | additional mental health support |
| | |
| Psychiatry on PBMU | A patient may be admitted to the Psychiatric team to continue |
| | eating disorder treatment if medically stable |
| | |
| Boost Plus | Nutritional supplement to replace meals for patients >= 11 yrs |
| | |
| BKE 1.5 | Boost Kids Essential – nutritional supplement for <11 yrs |
| | |
| Restoring Nutrition- | Patient education for caregivers, that explains what |
| Refeeding PE643 | to expect during hospital, stay and the different teams |
| | providing care to their child. |
| | |
| Support Nurse | Nurse with psychiatric and behavioral de-escalation |
| | Training who assists medical floor nurses as needed |
| | |
| GOC | Guideline of Care – information for nurses on how to care for |
| | patients with specific medical problems |
| | |
| Meal Support Classes | Training for caregivers provided by mental health providers in |
| | how to feed their child with an eating disorder |
| | |
| Psychiatry C&L | Psychiatry Consult & Liaison Service – team of |
| | psychiatrists and psychologists who provides consultation |
| | for hospitalized patients not in the psychiatric unit |
| | |
| PBMU Eating Disorder | Patients who are medically stable and admitted to the |
| Program | Psychiatric Service for eating disorder treatment |
| | |
| PCP | Primary care provider |
| | |
