# Supplementary material for: Implementation of Standardized Care for the Medical Stabilization of Patients With Anorexia Nervosa
Source: Pediatr Qual Saf. 2022 Aug 26;7(5):e582. doi: 10.1097/pq9.0000000000000582 (PMC9416762; doi:10.1097/pq9.0000000000000582)
Supplement: Supplementary file 5 [file pqs-7-e582-s005.pdf]

Figure 2. Patient Flow Pre- and Post-Pathway from Emergency Department to Admission to Discharge

Patient Status	Pre-Pathway	Intended Post-Pathway	Actual Post-Pathway
Evaluated in Emergency Department	x	x	x
Admitted to medical floor	x	x	x
Transferred to psychiatry unit as a medical patient	rare	x	rare to variable
Discharged from hospital	x	x	x
